# The Relationship Between Ostracism and Mental Health Issues Among College Students: The Chain Mediating Role of Rumination and Smartphone Addiction

**DOI:** 10.3390/bs16050769

**Published:** 2026-05-14

**Authors:** Chi Zhang, Zheng Wang, Wei Hua, Jiuchun Lin, Dongying Liu

**Affiliations:** 1 School of Chinese Language and Literature, Harbin Normal University, Harbin 150080, China; zhangchi@hrbnu.edu.cn; 2 School of Public Management, Guangzhou Nanfang College, Guangzhou 510970, China; 3 School of Education Science, Nanjing Normal University, Nanjing 210097, China; 02219@njnu.edu.cn; 4 Foreign Languages Studies, Faculty of Humanities and Social Sciences, Harbin Institute of Technology, Harbin 150001, China; 19940010@hit.edu.cn; 5 School of Literature, Heilongjiang University, Harbin 150080, China

**Keywords:** college students, ostracism, mental health issues, rumination, smartphone addiction

## Abstract

Mental health issues, particularly symptoms of anxiety and depression, have become increasingly prominent among college students, with negative experiences such as ostracism identified as important correlates. However, the potential pathways linking ostracism to mental health issues remain to be further explored. By incorporating rumination and smartphone addiction, this study aimed to examine the associations among ostracism, rumination, smartphone addiction, and mental health issues in college students. A cross-sectional, self-reported questionnaire survey was conducted among 632 college students, measuring these four variables simultaneously. Correlation analyses and mediation analyses using a chain mediation model with bootstrap estimation were employed to examine the relationships among these variables. The results revealed significant positive associations among ostracism, rumination, smartphone addiction, and mental health issues. Further analyses indicated that rumination and smartphone addiction played a chain mediating role between ostracism and mental health issues. The bootstrap confidence intervals confirmed the significance of the indirect effects, which accounted for 32.62% of the total effect. These findings provide insight into the interrelationships among ostracism, rumination, smartphone addiction, and mental health issues in college students, contributing to a better understanding of factors related to mental health issues in this population and offering a reference for future research and potential intervention development in college settings.

## 1. Introduction

Mental health issues are relatively common among college students and adversely affect their future development (Cleary et al., 2011; Cook, 2007; Soet & Sevig, 2006). Among these issues, anxiety and depression are the most prevalent, and previous studies have found that they frequently co-occur (Choi et al., 2020; Huang & Liu, 2023). As a result, prior research has often used instruments that assess both anxiety and depression, such as the Patient Health Questionnaire-4 (PHQ-4), as indicators of overall mental health status (Hua, 2025; Yan et al., 2024). A recent meta-analysis conducted in China indicates that mental health issues among college students have shown a significant upward trend over the past decade, with a particularly notable increase in depression and anxiety (Chen et al., 2022). Therefore, exploring the factors influencing college students’ mental health is of great significance. Existing research indicates that the emergence of mental health issues is influenced by multiple factors (Byrd & McKinney, 2012; Samuolis et al., 2015). Among these, ostracism, as a crucial social contextual factor, has been confirmed to be closely related to mental health issues in college students (Tariq et al., 2024). Following experiences of ostracism, college students are often more prone to engage in repetitive thinking about negative events. Furthermore, when facing real-life pressures, college students may frequently use smartphones to alleviate stress or escape from real-world situations, thereby increasing the risk of smartphone addiction (Dou & Feng, 2025). However, excessive use of smartphones not only fails to effectively relieve psychological distress but may also further exacerbate mental health issues. Taken together, these findings suggest that ostracism may influence mental health not only directly but also indirectly through a series of cognitive and behavioral responses. However, how these processes are systematically interconnected remains insufficiently understood. To address this gap, it is necessary to examine cognitive and behavioral mechanisms within an integrated framework. Existing evidence points to a potential sequential pathway that may help clarify these relationships. Specifically, ostracism may be initially associated with cognitive processing (e.g., rumination), which in turn is related to an increased reliance on behavioral coping strategies (e.g., smartphone addiction), ultimately linking to poorer mental health outcomes. Based on this reasoning, the present study proposes a chain mediation model to examine the roles of rumination and smartphone addiction in the relationship between ostracism and mental health issues among college students. This study aims to deepen the understanding of the relationship between ostracism and mental health issues among college students by identifying the key mediating mechanisms involved, thereby providing a theoretical basis for mental health interventions in higher education settings.

### 1.1. The Relationship Between Ostracism and Mental Health Issues

Ostracism is defined as being overlooked and excluded by other individuals or groups (Williams, 2007, 2009). According to the Temporal Need-Threat Model, ostracism threatens individuals’ fundamental psychological needs, including belonging, control, meaningful existence, and self-esteem, thereby impairing psychological functioning and triggering negative emotional responses (Williams, 2007, 2009). Empirical studies have also found that ostracism is closely associated with negative psychological symptoms such as depression and anxiety (Rudert et al., 2021; Zadro et al., 2006). For college students, the adverse effects of ostracism may be particularly pronounced. The college stage represents a critical transitional period in which individuals shift from adolescence to adult roles, accompanied by changes in social relationship structures, academic demands, and lifestyles (Kroshus et al., 2021; Shi et al., 2023). During this phase, college students may be more sensitive to peer acceptance and social evaluation. Simultaneously, they must cope with multiple developmental tasks, including academic pressure, employment stress, and family expectations, which may render them psychologically vulnerable (Brougham et al., 2009; Buizza et al., 2022). Under such circumstances, sustained ostracism tends to be associated with increased frustration of fundamental psychological needs, which in turn is associated with a heightened risk of mental health issues. Based on this reasoning, the present study proposes Hypothesis 1: Ostracism is significantly and positively associated with mental health issues among college students.

### 1.2. The Mediating Role of Rumination

Rumination may serve as a mediator in the relationship between ostracism and mental health issues among college students. Rumination is a way of responding to distress involving repetitive and passive focus on distress symptoms and their potential causes and consequences (Nolen-Hoeksema et al., 2008). Research indicates that when individuals experience negative events that are threatening or closely related to the self, such experiences can generate dissatisfaction in interpersonal relationships, accompanied by negative emotions and psychological distress (Borders & Liang, 2011; Nezlek et al., 2012). In this context, ostracism, as a quintessential negative social experience, may lead college students to repeatedly recall situations in which they were ostracized and continuously contemplate the causes and potential consequences of these events, thereby directing sustained attention and cognitive resources toward these experiences. According to Response Styles Theory, rumination, as a maladaptive response style, reinforces individuals’ negative cognitive processing and impairs their ability to effectively cope with problems, thereby prolonging and intensifying negative emotional experiences (Nolen-Hoeksema & Morrow, 1991; Nolen-Hoeksema et al., 2008). Furthermore, rumination may diminish individuals’ tendency to seek social support, making it more difficult for them to recover from negative events (Xia et al., 2022). Empirical studies have also demonstrated that rumination is closely associated with mental health issues such as depression and anxiety, suggesting that it may constitute a significant factor contributing to psychological impairment (Liu et al., 2023; Long & Yu, 2025; McLaughlin & Nolen-Hoeksema, 2011). Therefore, this study proposes Hypothesis 2: Rumination mediates the relationship between ostracism and mental health issues among college students.

### 1.3. The Mediating Role of Smartphone Addiction

In addition to rumination, smartphone addiction may also serve as a mediator in the relationship between college students’ ostracism and mental health issues. Smartphone addiction refers to the uncontrollable and excessive use of smartphones that interferes with fundamental activities in daily life and leads to negative consequences (Park & Lee, 2012; Sunday et al., 2021). College students face multiple pressures while simultaneously coping with complex social relationships, rendering them particularly sensitive to ostracism (K.-p. Li et al., 2024; Sun et al., 2023). Therefore, when individuals experience ostracism, they are more susceptible to negative emotions such as loneliness and may increase smartphone usage as a means of seeking emotional connection and psychological compensation. However, prolonged reliance on such compensatory usage patterns may gradually solidify smartphone use into habitual behavior, potentially escalating into smartphone addiction. For college students, smartphone addiction may not only reduce face-to-face interactions in real life but also contribute to academic procrastination and impaired sleep quality (Zhang & Wu, 2020; Zhao et al., 2025). These factors have been empirically demonstrated to be closely associated with mental health issues such as depression and anxiety (Alwhaibi & Al Aloola, 2023; X. Li et al., 2024; Yang et al., 2023). Based on the aforementioned analysis, this study proposes Hypothesis 3: Smartphone addiction mediates the relationship between college students’ ostracism and mental health issues.

### 1.4. The Chain Mediating Role of Rumination and Smartphone Addiction

Furthermore, a close association exists between rumination and smartphone addiction (Çorak, 2026; Peng et al., 2022). According to Compensatory Internet Use Theory, when individuals experience stressful events or negative emotional experiences in real life, they may turn to internet use as a means of compensating for unmet psychological needs in offline contexts, thereby obtaining emotional relief or psychological compensation (Kardefelt-Winther, 2014). In this process, due to smartphones’ high accessibility and immediate feedback features, they are more likely to become important tools for individuals to cope with negative emotions, consequently increasing the risk of developing dependence on these devices. For college students who experience ostracism, repeatedly dwelling on exclusionary experiences may further amplify negative emotions and exacerbate levels of rumination, subsequently prompting individuals to use smartphones as a means of temporarily escaping real-life distress and satisfying thwarted belongingness needs. However, although such compensatory usage patterns may alleviate negative emotions in the short term, they are prone to developing into dependent use over time and may adversely affect mental health. Based on this reasoning, the present study further proposes Hypothesis 4: Rumination and smartphone addiction play a chain mediating role in the relationship between ostracism and mental health issues among college students.

### 1.5. The Present Study

**Figure 1:**
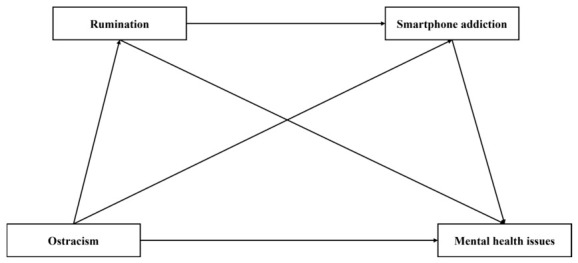
The hypothesized model.

In summary, this study constructs a chain mediation model (see Figure 1) to investigate the associations among college students’ ostracism, rumination, smartphone addiction, and mental health issues. Specifically, we propose a sequential pathway in which ostracism is positively associated with rumination, which in turn is positively associated with smartphone addiction; this, ultimately, is positively associated with mental health issues. This model helps to elucidate the potential psychological mechanisms underlying the relationship between ostracism and mental health among college students, thereby providing theoretical guidance and practical implications for mental health promotion and intervention efforts targeting ostracized populations in higher education settings.

## 2. Methods

### 2.1. Participants

This study employed a cross-sectional design. Participants were undergraduate students recruited from two private colleges in Guangdong Province. A total of 710 questionnaires were distributed. The questionnaires were administered on-site by researchers during students’ class breaks and collected uniformly. Prior to completion, participants were informed of the research purpose and instructions, with emphasis placed on the academic nature of the study and the assurance of anonymity. All students participated voluntarily and completed the questionnaires independently. Following data collection, the questionnaires were reviewed and screened; those with missing critical information or identical responses across all items were considered invalid and subsequently excluded. A total of 78 invalid questionnaires were removed, yielding 632 valid responses, with an effective response rate of 89.01%. Among the valid sample, there were 278 male participants and 354 female participants. The age range of the participants was 18 to 21 years, with a mean age of 18.54 years.

### 2.2. Measures

#### 2.2.1. Ostracism

Ostracism was measured using the Ostracism Experiences Scale (Carter-Sowell, 2010). This scale consists of 8 items rated on a 7-point Likert scale ranging from 1 (hardly ever) to 7 (almost always). Higher scores indicate more experiences of ostracism. The reliability of this scale has been established in Chinese samples (Yue et al., 2023). In the present study, Cronbach’s α coefficient for this scale was 0.963.

#### 2.2.2. Rumination

Rumination was assessed using the Ruminative Responses Scale (RRS) (Treynor et al., 2003). This scale comprises 10 items rated on a 4-point Likert scale ranging from 1 (almost never) to 4 (almost always). Higher scores reflect more severe rumination. The reliability of this scale has been validated in Chinese samples (Wang et al., 2026). In the present study, Cronbach’s α coefficient for this scale was 0.903.

#### 2.2.3. Smartphone Addiction

Smartphone addiction was measured using the scale developed by Roberts et al. (2014). This scale consists of 4 items rated on a 7-point Likert scale ranging from 1 (strongly disagree) to 7 (strongly agree). Higher scores indicate more severe smartphone addiction. The reliability of this scale has been established in Chinese samples (Dou & Feng, 2025). In the present study, Cronbach’s α coefficient for this scale was 0.855.

#### 2.2.4. Mental Health Issues

Mental health issues were assessed using The Patient Health Questionnaire-4 (PHQ-4) (Löwe et al., 2010). This scale comprises 4 items rated on a 4-point Likert scale ranging from 0 (not at all) to 3 (nearly every day). Higher scores reflect more severe mental health issues. The reliability of this scale has been validated in Chinese samples (Gao et al., 2023). In the present study, Cronbach’s α coefficient for this scale was 0.900.

### 2.3. Data Analysis

Data analysis was conducted using SPSS 26.0 software. First, Harman’s single-factor test was employed to examine common method bias. Subsequently, descriptive statistics and Pearson correlation analyses were performed to examine the relationships among the study variables. Prior to hypothesis testing, the assumptions of regression analysis were assessed. The results indicated that the Durbin–Watson statistic values were close to 2 and fell within the acceptable range, suggesting no serious autocorrelation. Additionally, all variance inflation factor (VIF) values were well below the commonly accepted threshold of 5, indicating that multicollinearity was not a concern. Furthermore, the absolute values of skewness and kurtosis for all variables were below 2, supporting the assumption of normality. To test the proposed mediation effects, Model 6 of the PROCESS macro for SPSS was used, employing a bias-corrected non-parametric percentile bootstrap method (Hayes, 2013). Bootstrap resampling was conducted with 5000 resamples to generate 95% confidence intervals for the indirect effects. An indirect effect was considered statistically significant if its 95% confidence interval did not include zero.

## 3. Results

### 3.1. Common Method Bias Test

As all data in this study were collected using questionnaire methods, which may potentially introduce common method bias (Podsakoff et al., 2003), Harman’s single-factor test was conducted to examine this issue. The results of the unrotated factor analysis revealed that five factors had eigenvalues greater than 1. The total variance explained by the first factor was below the critical threshold of 40%, indicating that common method bias was not a serious concern in this study.

### 3.2. Descriptive Statistics and Correlation Analysis

As presented in Table 1, ostracism, rumination, smartphone addiction, and mental health issues were all positively correlated with each other.

### 3.3. Mediation Effect Test

Data analysis was conducted using the PROCESS macro for SPSS developed by Hayes. With the exception of gender, all variables in the model were standardized. With gender entered as a control variable, ostracism was specified as the independent variable, mental health issues as the dependent variable, and rumination and smartphone addiction as mediating variables. PROCESS Model 6 was selected to examine the chain mediating effects.

**Figure 2:**
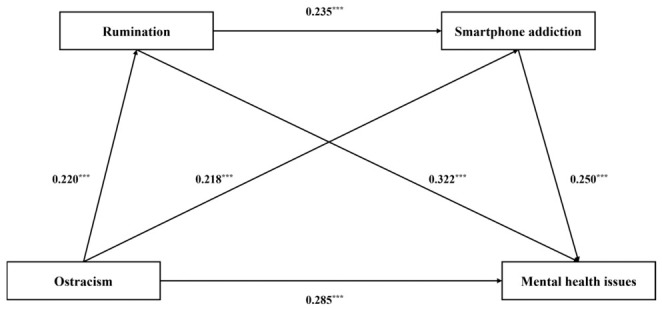
The final chain mediation model. *** *p* < 0.001.

The results of the regression analysis are presented in Table 2 and Figure 2. Ostracism was significantly and positively associated with mental health issues (*β* = 0.423, *p* < 0.001, 95% CI [0.353, 0.494]) and rumination (*β* = 0.220, *p* < 0.001, 95% CI [0.145, 0.296]). Both ostracism (*β* = 0.218, *p* < 0.001, 95% CI [0.143, 0.293]) and rumination (*β* = 0.235, *p* < 0.001, 95% CI [0.159, 0.310]) were significantly and positively associated with smartphone addiction. Furthermore, when ostracism (*β* = 0.285, *p* < 0.001, 95% CI [0.220, 0.351]), rumination (*β* = 0.322, *p* < 0.001, 95% CI [0.255, 0.388]), and smartphone addiction (*β* = 0.250, *p* < 0.001, 95% CI [0.183, 0.317]) were simultaneously entered into the model, all three variables were positively associated with mental health issues.

The total indirect effect accounted for 32.62% of the total effect. The mediation effect analysis results are presented in Table 3. Specifically, the mediating effects consisted of three indirect pathways: Indirect Effect 1 (0.071) through “ostracism → rumination → mental health issues”; Indirect Effect 2 (0.055) through “ostracism → smartphone addiction → mental health issues”; and Indirect Effect 3 (0.013) through “ostracism → rumination → smartphone addiction → mental health issues.” The 95% confidence intervals for all three indirect effects did not include zero, indicating that all indirect effects were statistically significant.

## 4. Discussion

Given the increasing prominence of mental health issues among college students, the present study examined the association between ostracism and mental health issues in a sample of Chinese college students. Furthermore, this study investigated the chain mediating roles of rumination and smartphone addiction in the relationship between college students’ ostracism and mental health issues. The findings provide important empirical support for interventions targeting college students’ mental health issues within the context of a digital environment.

First, the results of this study revealed a positive association between ostracism and mental health issues among college students, thereby supporting Hypothesis 1. This finding suggests that ostracism, as a significant social stressor, is negatively associated with individuals’ psychological well-being. Specifically, when individuals encounter ostracism in social interactions, their need for belonging is readily threatened, which in turn is associated with negative emotions such as loneliness (Ren et al., 2021; Sakız et al., 2021). If the negative emotions elicited by ostracism experiences remain unrelieved over extended periods, individuals may tend to adopt avoidant or withdrawn interpersonal coping strategies, thereby reducing social engagement (Lei et al., 2024). Such diminished social interaction may be associated with their weakened capacity to cope with stress, ultimately linking to an increased risk of mental health issues, including depression and anxiety. Therefore, in college mental health education, attention should be directed toward social stressors such as ostracism. Practical efforts should focus on implementing targeted support strategies (e.g., peer support or counseling) for students who experience sustained ostracism, particularly when such experiences are identified through self-reports or routine screening in campus mental health services.

Second, our findings demonstrated that rumination mediated the relationship between ostracism and mental health issues among college students, supporting Hypothesis 2. This finding suggests that when college students experience ostracism, their mental health is associated not only with external events but also with their cognitive processing. Specifically, as a significant stressful context, ostracism is associated with a greater tendency to dwell repeatedly on the negative experience, which in turn is associated with elevated levels of rumination (Jiang & Poon, 2023). In this state, individuals’ attention tends to become excessively focused on negative emotions and their causes, while they are less likely to employ effective problem-solving strategies to cope with these stressors (Watkins & Roberts, 2020). Consequently, when college students experience elevated levels of rumination following ostracism, their mental health is associated with greater vulnerability to impairment. The findings of this study suggest that interventions targeting rumination may be beneficial in mitigating the negative correlates of ostracism among college students. Colleges could implement workshops aimed at helping students identify rumination, for example, through mindfulness-based practices. In addition, campus counseling services may incorporate specific strategies such as attention-shifting exercises when supporting students who report experiences of ostracism, thereby reducing the persistence of negative thought patterns associated with ostracism.

Third, the results of this study indicated that ostracism was positively correlated with smartphone addiction among college students, which in turn was positively correlated with mental health issues, thereby supporting Hypothesis 3. This finding aligns with existing research suggesting that ostracism is positively associated with problematic behaviors such as smartphone addiction (Yue et al., 2022). When individuals experience ostracism, they are prone to developing negative emotions and tendencies toward escapism, rendering them more inclined to seek emotional compensation and solace through virtual spaces (Poon, 2018). In this process, as smartphones serve as important mediums for accessing online social interaction and entertainment, both the frequency of use and degree of dependence may progressively increase, and this pattern is positively associated with a higher risk of smartphone addiction. Notably, smartphone addiction not only represents a behavioral dependence but may also be associated with disruptions to college students’ study and leisure time, which in turn is correlated with mental health issues such as depression and anxiety (Shang et al., 2024; Volungis et al., 2020). Therefore, this study further elucidates the potential mechanism through which ostracism is associated with college students’ mental health from the perspective of smartphone addiction. The findings also suggest that, when considering the correlates of ostracism in relation to college students’ mental health, targeted interventions might benefit from focusing on reducing problematic smartphone use as a key factor. Specifically, colleges and counseling services may enhance students’ awareness of maladaptive smartphone use and combine this with relevant strategies (e.g., screen time management and digital literacy programs).

Finally, our findings indicated that rumination and smartphone addiction jointly functioned as a chain mediating mechanism in the association between ostracism and mental health issues among college students, thereby supporting Hypothesis 4. This result highlights not only the close association between rumination and smartphone addiction but also how ostracism may be linked to mental health through interconnected cognitive and behavioral pathways within a specific cultural context. In China’s collectivist culture, which emphasizes a sense of belonging to a group, individuals often place greater importance on interpersonal relationships (He et al., 2024). In this context, experiences of ostracism are associated with stronger cognitive and emotional responses, which in turn are related to a higher tendency for repetitive negative thinking about the ostracism experience (Wesselmann et al., 2013). At the same time, academic and employment pressures, along with strong peer comparison norms, are further associated with increased ruminative tendencies. Under such circumstances, individuals may be more inclined to rely on smartphones as an accessible tool for immediate psychological compensation. Although this behavior may be associated with temporary relief from ostracism, it may also correlate with higher levels of problematic or addictive smartphone use (Sun et al., 2023). Over time, this pattern may be linked to disruptions in daily functioning and real-life social interactions, which in turn may be associated with a higher risk of mental health issues such as depression and anxiety among college students.

## 5. Limitations

Several limitations of this study should be noted. First, due to the cross-sectional design employed, the observed associations among variables cannot be interpreted as causal, and the directionality of these relationships remains tentative. It is also possible that reverse or bidirectional relationships exist; for example, individuals with more pronounced mental health issues may be more likely to perceive ostracism or to increase smartphone use as a coping strategy. Therefore, future research is encouraged to adopt longitudinal designs to further examine and validate the proposed chain mediation model. Second, the sample was primarily drawn from two private colleges in Guangdong Province, and only basic demographic information (i.e., gender and age) was collected, with no further disciplinary characteristics available. This may limit the contextual specificity and generalizability of the findings. Future studies could expand the sample size and enhance its representativeness. Third, the reliance on self-reported data may introduce social desirability bias or common method bias. To address this limitation, future research could adopt a multi-method approach. For example, ostracism could be assessed using experimental paradigms (e.g., the Cyberball paradigm), and smartphone addiction could be measured using objective smartphone usage data (e.g., screen time). Finally, other contextual and individual-level factors not included in the present model, such as personality traits, may also influence these associations, potentially limiting the comprehensiveness of the proposed model. Future research could incorporate these variables to develop a more comprehensive model.

## 6. Conclusions

In conclusion, by establishing a chain mediation model, this study provides a novel perspective for the existing literature and contributes to our understanding of the associations between ostracism and mental health issues among college students. The findings indicate that rumination and smartphone addiction play a chain mediating role in the association between ostracism and mental health issues. Specifically, ostracism is associated with increased levels of rumination, which in turn are associated with higher levels of smartphone addiction; such behaviors are further associated with mental health issues. This model not only elucidates the multiple psychological processes that may be involved in mental health difficulties among college students experiencing ostracism but also offers a theoretical foundation and practical reference for future mental health interventions in college settings.

## Figures and Tables

**Table 1:** Descriptive statistics and correlation analysis of each variable.

Variable	*M*	*SD*	1	2	3	4
1. Ostracism	18.633	9.013	1			
2. Rumination	24.671	5.803	0.224 **	1		
3. Smartphone addiction	17.342	5.107	0.267 **	0.271 **	1	
4. Mental health issues	4.119	2.268	0.424 **	0.454 **	0.413 **	1

Note: *M* is mean value; *SD* is standard deviation. ** *p* < 0.01.

**Table 2:** Regression analysis of the relationship between variables.

Dependent Variable	Independent Variable	*β*	*t*	Bootstrap 95% CI	*R* ^2^	*F*
Mental health issues	Gender	−0.035	−0.481	[−0.178, 0.108]	0.180	69.104 ***
	Ostracism	0.423	11.722 ***	[0.353, 0.494]		
Rumination	Gender	−0.218	−2.807 **	[−0.371, −0.066]	0.062	20.810 ***
	Ostracism	0.220	5.706 ***	[0.145, 0.296]		
Smartphone addiction	Gender	0.208	2.749 **	[0.059, 0.356]	0.129	31.003 ***
	Ostracism	0.218	5.710 ***	[0.143, 0.293]		
	Rumination	0.235	6.099 ***	[0.159, 0.310]		
Mental health issues	Gender	−0.004	−0.059	[−0.131, 0.123]	0.370	92.081 ***
	Ostracism	0.285	8.547 ***	[0.220, 0.351]		
	Rumination	0.322	9.546 ***	[0.255, 0.388]		
	Smartphone addiction	0.250	7.357 ***	[0.183, 0.317]		

Note: ** *p* < 0.01; *** *p* < 0.001.

**Table 3:** The mediation effect analysis.

Effect Type	Effect Size	SE	Bootstrap 95% CI
Total effect	0.423	0.036	[0.353, 0.494]
Direct effect	0.285	0.033	[0.220, 0.351]
Indirect effect 1	0.071	0.017	[0.040, 0.107]
Indirect effect 2	0.055	0.014	[0.030, 0.083]
Indirect effect 3	0.013	0.004	[0.006, 0.022]

Note: Indirect effects 1: Ostracism → Rumination → Mental Health Issues; Indirect effects 2: Ostracism → Smartphone addiction → Mental Health Issues; Indirect effects 3: Ostracism → Rumination → Smartphone addiction → Mental Health Issues.

## Data Availability

The data are available from the corresponding author upon reasonable request.
